# A chromosome-level genome assembly for the dugong (*Dugong dugon*)

**DOI:** 10.1093/jhered/esae003

**Published:** 2024-01-20

**Authors:** Dorothy Nevé Baker, Linelle Abueg, Merly Escalona, Katherine A Farquharson, Janet M Lanyon, Diana Le Duc, Torsten Schöneberg, Dominic Absolon, Ying Sims, Olivier Fedrigo, Erich D Jarvis, Katherine Belov, Carolyn J Hogg, Beth Shapiro

**Affiliations:** Department of Ecology and Evolutionary Biology, University of California Santa Cruz, Santa Cruz, CA, United States; Vertebrate Genome Laboratory, The Rockefeller University, New York, NY, United States; Department of Biomolecular Engineering, University of California Santa Cruz, Santa Cruz, CA, United States; Faculty of Science, School of Life and Environmental Sciences, The University of Sydney, Sydney, NSW, Australia; Australian Research Council Centre of Excellence for Innovations in Peptide and Protein Science, The University of Sydney, NSW, Australia; School of Biological Sciences, The University of Queensland, St Lucia, QLD, Australia; Institute of Human Genetics, University Medical Center Leipzig, Leipzig, Germany; Medical Faculty, Rudolf Schönheimer Institute of Biochemistry, University of Leipzig, Leipzig, Germany; School of Medicine, University of Global Health Equity, Kigali, Rwanda; Wellcome Sanger Institute, Wellcome Genome Campus, Hinxton, United Kingdom; Wellcome Sanger Institute, Wellcome Genome Campus, Hinxton, United Kingdom; Colossal Biosciences, Cambridge, MA, United States; Vertebrate Genome Laboratory, The Rockefeller University, New York, NY, United States; Howard Hughes Medical Institute, Chevy Chase, MD, United States; Faculty of Science, School of Life and Environmental Sciences, The University of Sydney, Sydney, NSW, Australia; Australian Research Council Centre of Excellence for Innovations in Peptide and Protein Science, The University of Sydney, NSW, Australia; Faculty of Science, School of Life and Environmental Sciences, The University of Sydney, Sydney, NSW, Australia; Australian Research Council Centre of Excellence for Innovations in Peptide and Protein Science, The University of Sydney, NSW, Australia; Department of Ecology and Evolutionary Biology, University of California Santa Cruz, Santa Cruz, CA, United States; Howard Hughes Medical Institute, Chevy Chase, MD, United States

**Keywords:** conservation, long-read assembly, marine mammals, sirenians, Vertebrate Genomes Project, whole genome

## Abstract

The dugong (*Dugong dugon*) is a marine mammal widely distributed throughout the Indo-Pacific and the Red Sea, with a Vulnerable conservation status, and little is known about many of the more peripheral populations, some of which are thought to be close to extinction. We present a de novo high-quality genome assembly for the dugong from an individual belonging to the well-monitored Moreton Bay population in Queensland, Australia. Our assembly uses long-read PacBio HiFi sequencing and Omni-C data following the Vertebrate Genome Project pipeline to reach chromosome-level contiguity (24 chromosome-level scaffolds; 3.16 Gbp) and high completeness (97.9% complete BUSCOs). We observed relatively high genome-wide heterozygosity, which likely reflects historical population abundance before the last interglacial period, approximately 125,000 yr ago. Demographic inference suggests that dugong populations began declining as sea levels fell after the last interglacial period, likely a result of population fragmentation and habitat loss due to the exposure of seagrass meadows. We find no evidence for ongoing recent inbreeding in this individual. However, runs of homozygosity indicate some past inbreeding. Our draft genome assembly will enable range-wide assessments of genetic diversity and adaptation, facilitate effective management of dugong populations, and allow comparative genomics analyses including with other sirenians, the oldest marine mammal lineage.

## Introduction

**Fig. 1.**
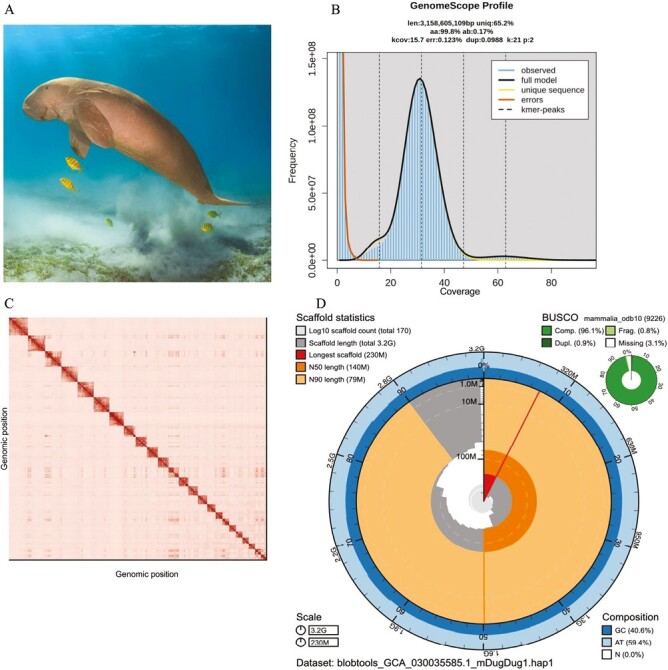
Dugong high-quality reference assembly. A) An adult dugong. Margarita Granovskaya via stock.adobe.com B) K-mer spectrum output generated from adapter-filtered PacBio HiFi data using GenomeScope 2.0. The bimodal pattern observed corresponds to a diploid genome. K-mers covered at lower coverage and lower frequency correspond to differences between haplotypes, and the higher coverage and higher frequency k-mers correspond to the similarities between haplotypes. C) Omni-C Contact maps for the curated genome assembly of haplotype 1 generated with PretextSnapshot. Omni-C contact maps translate proximity of genomic regions in 3D space to contiguous linear organization. Each cell in the contact map corresponds to sequencing data supporting the linkage (or join) between two of such regions. Scaffolds are separated by black lines. D) BlobToolKit Snail plot showing a graphical (*continued next page*) representation of the quality metrics presented in Table 2 for the *Dugong dugon* assembly for haplotype 1 (mDugdug1.hap1). The plot circle represents the full size of the assembly. From the inside out, the central plot covers length-related metrics. The red line represents the size of the longest scaffold; all other scaffolds are arranged in size order moving clockwise around the plot and drawn in gray starting from the outside of the central plot. Dark and light orange arcs show the scaffold N50 and scaffold N90 values. The central light gray spiral shows the cumulative scaffold count with a white line at each order of magnitude. White regions in this area reflect the proportion of Ns in the assembly. The dark versus light blue area around it shows mean, maximum, and minimum GC versus AT content at 0.1% intervals. The legend in the lower left indicates the scale of the circumference (3.2G total assembly size) and radius (230M longest scaffold) of the main plot. A summary of complete, fragmented, duplicated, and missing BUSCO genes in the mammalia_odb10 set is shown in the top right (Challis et al. 2020).

Dugongs (*Dugong dugon*; Fig. 1A) are marine mammals with a broad but fragmented distribution throughout the Indian and western Pacific Oceans (Husar 1978). Dugongs belong to the order Sirenia along with manatees, and are the only extant representative of the family Dugongidae. They are also the closest relative of the Steller’s sea cow, a giant sirenian that was hunted to extinction in the 18th century. Dugongs prefer shallow coastal waters and are mainly herbivorous, relying on seagrass meadows for both food and habitat (Best 1981). Dugongs are a culturally important species to Torres Strait Islander and many coastal Aboriginal communities for cultural ceremonies, hunting, and in custodianship of Sea Country (Leong 1998; Lincoln et al. 2021). Little is published in the literature about dugong behavior—their shy and elusive nature makes them challenging to study in the wild and, unlike many other small marine mammals, they are difficult to maintain in captivity (Bertram and Bertram 1973; Goto et al. 2004). While some areas, such as northern and eastern Australia, have robust ecological monitoring programs for dugongs and co-management programs with Indigenous communities (Tibbetts et al. 2019; Lincoln et al. 2021; Cleguer et al. 2023), other dugong populations throughout south Asia and eastern Africa are data deficient (Marsh et al. 2002). The IUCN lists dugongs as Vulnerable; however, some populations are thought to be close to extinction due primarily to habitat destruction and fisheries bycatch (Marsh et al. 1995, 2002). Evidence from aerial surveys, habitat mapping, and interviews with local communities suggests that the global range of dugongs has contracted (Marsh et al. 2002), leaving potentially endangered and isolated relict populations—particularly in the western Indian Ocean—and generating concern about loss of genetic diversity (Plön et al. 2019). However, substantial uncertainty remains concerning the global status of dugongs.

Many questions remain relating to dugong demographics, movement, and population structure that can be addressed using whole-genome data. Previous genetic studies have relied primarily on analyzing the distribution of mitochondrial control region haplotypes (Blair et al. 2014; Plön et al. 2019; Srinivas et al. 2020; Garrigue et al. 2022). These studies have shown that dugong mitochondrial haplotypes show significant geographic structure throughout their range and generally high mitochondrial haplotype diversity range-wide (Blair et al. 2014; Seddon et al. 2014; Plön et al. 2019), with lower diversity at the range periphery (Plön et al. 2019; Garrigue et al. 2022). Microsatellite and SNP genotypes also recovered significant geographic structure as well as isolation by distance, reflecting generally low dispersal among dugongs (Seddon et al. 2014; Cope et al. 2015; McGowan et al. 2023). The environmental forces contributing to this structure are not fully understood; however, sea level fluctuations associated with Pleistocene glacial cycles may have allowed range expansion and contraction by repeatedly creating and destroying the shallow near-shore seagrass habitat upon which dugongs rely (Woodruff 2010). For example, much of the marine near-shore environment around northern Australia and southeast Asia—the approximate geographic center of present-day dugong range—was not submerged until the end of the last glacial maximum 17,000 yr ago (Ludt and Rocha 2015). Cryptic marine barriers (e.g. tidal and current patterns) and breaks in seagrass habitat may also play a role (McGowan et al. 2023).

Here, we present a highly contiguous, chromosome-level de novo high-quality genome assembly for the dugong, along with initial estimates of genomic diversity and demographic history. Our assembly provides a resource for future genomic studies of dugong population structure, conservation status, and evolutionary history, and will contribute to the larger Vertebrate Genome Project (Rhie et al. 2021). Along with existing draft-quality genome assemblies for manatees and the extinct Steller’s sea cow, this assembly will also allow future comparative studies of sirenians and other marine mammals.

## Methods

### Biological materials

The sample was collected from a wild adult female dugong captured as part of an ongoing research program in Moreton Bay, Queensland, Australia (−27.15148032, 153.0415985) on 17 May 2022. A total volume of 16 ml of whole blood in EDTA was collected nonlethally and immediately flash frozen in liquid nitrogen and stored at −80 °C until genomic DNA extraction. Samples were collected under Scientific Purposes Permit # WA0019236, Moreton Bay Marine Park permit # MPP18-001119, and UQ Animal Ethics permit # 2021/AE000821.

### Nucleic acid extraction

We isolated high molecular weight (HMW) genomic DNA (>40 kbp) using a Circulomics Nanobind CBB kit (Pacific Biosciences—PacBio, Cat. #102-207-600). Prior to library preparation, the genomic DNA was pretreated for damage using the NEBNext FFPE DNA Repair Mix (New England Biolabs, Massachusetts), according to the manufacturer’s instructions.

### PacBio HiFi library preparation and sequencing

Two HiFi SMRTbell libraries were constructed using the SMRTbell Express Template Prep Kit v2.0 (PacBio, Cat. #100-938-900) according to the manufacturer’s instructions. HMW gDNA was sheared to a target DNA size distribution between 15 and 20 kbp. The sheared gDNA was concentrated using 0.45× of AMPure PB beads (PacBio, Cat. #100-265-900) for the removal of single-strand overhangs at 37 °C for 15 min, followed by further enzymatic steps of DNA damage repair at 37 °C for 30 min, end repair and A-tailing at 20 °C for 10 min and 65 °C for 30 min, ligation of overhang adapter v3 at 20 °C for 60 min and 65 °C for 10 min to inactivate the ligase, then nuclease treated at 37 °C for 1 h. The SMRTbell library was purified and concentrated with 0.45× Ampure PB beads (PacBio, Cat. #100-265-900) for size selection using the BluePippin/PippinHT system (Sage Science, Massachusetts; Cat. #BLF7510/HPE7510) to collect fragments greater than 7 to 9 kbp. The 15 kbp average HiFi SMRTbell libraries were sequenced at the Australian Genome Research Facility in the University of Queensland using 3 8M SMRT cells, Sequel II sequencing chemistry 2.0, and 30-h movies each on a PacBio Sequel II sequencer.

### Omni-C library preparation and sequencing

The Omni-C library was prepared from 3 ml of frozen blood using Dovetail Omni-C Kit (Dovetail Genomics, California) according to the manufacturer’s Mammalian protocol v1.4 with minor modifications. In brief, cells were isolated from thawed blood and chromatin fixed in place in the nucleus. Fixed chromatin was digested with DNase I then extracted and digestion profiles were assessed using TapeStation D5000 screen tapes (Agilent Technologies, California). Chromatin ends were repaired and ligated to a biotinylated bridge adapter followed by proximity ligation of adapter-containing ends. After proximity ligation, crosslinks were reversed and the DNA purified from proteins. Purified DNA was treated to remove biotin that was not internal to ligated fragments. An NGS library was generated using an NEB Ultra II DNA Library Prep kit (New England Biolabs, Massachusetts) with an Illumina-compatible y-adaptor. Biotin-containing fragments were then captured using streptavidin beads. The post-capture product was split into two replicates prior to PCR enrichment to preserve library complexity with each replicate receiving unique dual indices. The libraries were then sequenced at the Ramaciotti Center for Genomics at the University of New South Wales (Sydney, Australia) on an Illumina NextSeq 500 platform to generate approximately 100 million 2 × 150 bp read pairs per Gbp genome size.

### Nuclear genome assembly

We assembled the dugong genome following the Vertebrate Genomes Project (VGP) v2.0 Galaxy assembly pipeline (Table 1, see Data availability statement for link to all assembly scripts) (Rhie et al. 2021; Larivière et al. 2023). In particular, we removed remnant adapter sequences from the PacBio HiFi dataset using cutadapt (Martin 2011) and used them to generate the initial phased diploid contigs using HiFiasm in HiC mode, with Omni-C used to phase the haplotypes (Cheng et al. 2021). We scaffolded both contig haplotypes using the Omni-C data with YaHS (Zhou et al. 2023). We generated Omni-C contact maps for both assemblies by aligning the Omni-C data against the corresponding assembly with BWA-MEM (Li 2013). We identified ligation junctions, and merged alignments using the Arima mapping pipeline (https://github.com/ArimaGenomics/mapping_pipeline) implemented as bellerophon in Galaxy (Kerkvliet et al. 2019). We then performed manual curation on haplotype 1 to correct structural errors, improve contiguity, and name chromosomes following Howe et al. (2021). To do so, we used the PretextSuite (https://github.com/wtsi-hpag/PretextView; https://github.com/wtsi-hpag/PretextMap; https://github.com/wtsi-hpag/PretextSnapshot) to visualize the contact maps and checked for major misassemblies and cut the assemblies at the closest joins where the misassemblies were found. We then checked for contamination using the BlobToolKit Framework (Challis et al. 2020). Finally, we trimmed remnants of sequence adaptors identified during NCBI contamination screening.

**Table 1.** Assembly pipeline and software used.

	Software and options	Version
**Assembly**		
Filtering PacBio HiFi adapters	cutadapt -j=32 -b ATCTCTCTCAACAACAACAACGGAGGAGGAGG AAAAGAGAGAGAT -b ATCTCTCTCTTTTCCTCCTCCTCCGTTGTTGTTG TTGAGAGAGAT --output=out1.fq.gz --error rate=0.1 --times=1 --overlap=3 --action=trim --revcomp --discard-trimmed	4.0+galaxy0
K-mer counting	Meryl (*k* = 21)	1.3+galaxy4
Estimation of genome size and heterozygosity	GenomeScope	2.0+galaxy1
De novo assembly (contiging)	hifiasm in HiC mode: hifiasm -t 32 -o output -f 37 -l 3 -s 0.75 -O 1 --l-msjoin 500000 --primary	0.16.1+galaxy3
Omni-C scaffolding	yahs --no-mem-check	1.2a.2+galaxy0
**Omni-C contact map generation**		
Short-read alignment	BWA-MEM2	2.2.1+galaxy0
SAM/BAM processing and filtering	Arima mapping pipeline (implemented as bellerophon)	1.0+galaxy0
Contact map visualization	PretextMap	1.0+galaxy0
	PretextSnapshot	0.0.3
**Organelle assembly**		
Mitogenome assembly	mitohifi.py -f AY075116.1.fasta -g AY075116.1.gb -p 70 -t 32 -o 2	2
**Genome quality assessment**		
Basic assembly metrics	gfastats	1.3.0+galaxy0
Assembly completeness	BUSCO (-m geno, -l vertebrata/mammalia)	5.3.2+galaxy0
	Merqury	1.3+galaxy2
**Contamination screening**		
Local alignment tool	Blast+	2.14.0
General contamination screening	BlobToolKit	4.1.7
**Comparison to *E. maximus***		
Sequence alignment	nucmer (mummer)	3.9.4alpha
**Diversity and demographic history**		
Runs of homozygosity detection	ROHan	
Effective population size fluctuations	PSMC -N25 -t15 -r5 -p 4 + 25*2 + 4+6	0.6.5-r67

Software citations are listed in the text.

**Table 2.** Sequencing and assembly statistics, and accession numbers.

BioProjects and vouchers	VGP NCBI BioProject			PRJNA489243			
	Species NCBI BioProject			PRJNA970804			
	NCBI BioSample			SAMN33212336			
	NCBI Genome accessions			Haplotype 1		Haplotype 2	
	Assembly accession			GCA_030035585.1		GCA_030020955.1	
	Genome sequences			JASCZL000000000		JASCZM000000000	
Genome sequence	PacBio HiFi reads	Run		3 PACBIO_SMRT (Sequel II) runs: 6.5 million reads, 102 Gbases			
	Omni-C Illumina reads	Run		2 ILLUMINA (Illumina NovaSeq 6000) runs: 457.5 million reads, 138.2Gb			
	Assembly identifier (quality code)^a^			mDugDug1 1(8.8.P8.Q70.C99)			
	HiFi read coverage^b^			32.0X			
Genome Assembly Quality Metrics				Haplotype 1		Haplotype 2	
	Number of contigs			294		256	
	Contig N50 (bp)			57,632,671		57,883,746	
	Contig NG50 (bp)			57,632,671		57,883,746	
	Longest contigs			162,184,114		209,448,431	
	Number of scaffolds			198		167	
	Scaffold N50 (bp)			177,379,183		138,031,769	
	Scaffold NG50 (bp)			177,379,183		138,031,769	
	Largest scaffold			267,865,978		230,272,189	
	Size of final assembly (bp)			3,159,179,246		3,154,861,630	
	Phased block NG50 (bp)			57,632,671		57,883,746	
	Gaps per Gbp (# Gaps)			25 (79)		28 (88)	
	Indel QV (frameshift)			41.52		42.16	
	Base pair QV			70.4553		70.3254	
				Full assembly = 70.3899			
	K-mer completeness			97.9001		97.8847	
				Full assembly = 99.7025			
	BUSCO completeness (vertebrata), *n* = 3354		C^c^	S^c^	D^c^	F^c^	M^c^
	Vertebrata *n* = 3354	H1^d^	97.9%	95.9%	2.0%	1.0%	1.1%
		H2^d^	97.8%	95.7%	2.1%	1.1%	1.1%
	Mammalia *n* = 9226	H1^d^	96.2%	95.3%	0.9%	0.8%	3.0%
		H2^d^	96.1%	95.2%	0.9%	0.8%	3.1%
	Organelles			1 complete mitochondrial sequence (pending NCBI accession code)			

^a^Assembly quality code *x*·*y*·*P*·*Q*·*C* derived notation, from (Rhie et al. 2021). *x* = log_10_[contig NG50]; *y* = log_10_[scaffold NG50]; *P* = log_10_ [phased block NG50]; *Q* = Phred base accuracy QV (Quality value); *C* = % genome represented by the first “n” scaffolds, following a karyotype of 2n = 48 inferred from ancestral taxa *Trichechus manatus* (Noronha et al. 2022).

^b^Read coverage and NGx statistics have been calculated based on the estimated genome size of 3.16 Gbp.

^c^Complete BUSCOs (C), Complete and single-copy BUSCOs (S), Complete and duplicated BUSCOs (D), Fragmented BUSCOs (F), Missing BUSCOs (M).

^d^(H1) Haplotype 1 and (H2) Haplotype 2 assembly values.

To identify the X chromosome from draft chromosome assignments, we aligned our genome (mDugDug1.hap1) to the annotated genome assembly for the Indian elephant *Elephas maximus indicus* (Vertebrate Genome Project, GenBank Accession GCA_024166365.1) using nucmer (Marçais et al. 2018), as this was the closest dugong relative with a chromosome-level assembly available.

### Mitochondrial genome assembly

We assembled the mitochondrial genome of the dugong from the PacBio HiFi reads using the reference-guided pipeline MitoHiFi (https://github.com/marcelauliano/MitoHiFi) (Uliano-Silva et al. 2023). A previously assembled dugong mitogenome (NCBI:AY075116.1) was used as the starting reference sequence. After completion of the nuclear genome, we searched for matches of the resulting mitochondrial assembly sequence in the nuclear genome assembly using BLAST+ (Camacho et al. 2009) and filtered out contigs and scaffolds from the nuclear genome with a percentage of sequence identity >99% and size smaller than the mitochondrial assembly sequence. We annotated the resulting mitochondrial assembly using GeSeq (Tillich et al. 2017), implementing the tRNAscan-SE v.2.0.7 3rd party tRNA annotator with the vertebrate mitochondrial tRNA database for tRNA annotation.

### Genome size estimation and quality assessment

We generated k-mer counts from the PacBio HiFi reads using meryl (https://github.com/marbl/meryl). We then applied GenomeScope 2.0 (Ranallo-Benavidez et al. 2020) to the k-mer database to estimate genome features including genome size, heterozygosity, and repeat content. To evaluate genome quality and completeness we used BUSCO (Manni et al. 2021) with both the vertebrate ortholog database (vertebrata_odb10) which contains 3,354 genes and the mammalian ortholog database (mammalia_odb10) which contains 9,226 genes. Assessment of base level accuracy (QV) and k-mer completeness was performed using the previously generated meryl database and merqury (Rhie et al. 2021). To obtain general contiguity metrics, we ran gfastats (Gurevich et al. 2013). We further estimated genome assembly accuracy via BUSCO gene set frameshift analysis using the pipeline described in Korlach et al. (2017) with the mammalian database. Measurements of the size of the phased blocks are based on the size of the contigs generated by HiFiasm in HiC mode (initial diploid assembly).

Following the quality metrics nomenclature established by Rhie et al. (2021), we used the derived genome quality notation *x*·*y*·*P*·*Q*·*C*, where *x* = log_10_[contig NG50]; *y* = log_10_[scaffold NG50]; *P* = log_10_[phased block NG50]; *Q* = Phred base accuracy QV (quality value); *C* = % genome represented by the first “n” scaffolds, following a karyotype of 2n = 48 inferred from ancestral taxa *Trichechus manatus manatus* (Noronha et al. 2022). Quality metrics for the notation were calculated on the primary assembly.

### Diversity and demographic history

We used ROHan (Renaud et al. 2019) on the filtered and aligned Omni-C data to refine estimates of genome-wide heterozygosity and identify runs of homozygosity (ROH), indicative of inbreeding. We applied the pairwise sequentially Markovian coalescent (PSMC) (Li and Durbin 2011) approach to infer historical effective population size of dugongs over time. We generated a diploid consensus sequence using the mpileup function of SAMtools (v0.1.18; with “-C50” option), bcftools to call variants, and available scripts from PSMC package to convert file formats. We required that sequencing depth for each locus was above one-third of average coverage (“-d” option) and less than twice of average coverage (“-D” option), and that consensus base quality was above Q20. We ran PSMC using the recommended parameters (Table 1) and 100 rounds of bootstrapping. We scaled our estimates using the previously reported dugong generation time of 27 yr (McDonald 2005) and a mutation rate of 6.25e-9 mutations per nucleotide per generation, calculated using the divergence rate between dugongs and Steller’s sea cows (Le Duc et al. 2022).

## Results

The PacBio HiFi and Omni-C sequencing libraries generated 6.5 million reads and 457.5 million read pairs, respectively. The PacBio HiFi reads yielded a mean read length of 15,629 bp and 32-fold coverage based on the GenomeScope 2.0 genome size estimation of 3.16 Gbp. From the same software and HiFi reads, we estimated 0.123% sequencing error rate and 0.211% nucleotide heterozygosity rate. The k-mer spectrum based on PacBio HiFi reads shows a slightly bimodal distribution with 2 peaks at ~18- and ~32-fold coverage (Fig. 1B), where peaks correspond to heterozygous and homozygous states of a diploid species.

The final assembly (mDugDug1) consists of two haplotypes (haplotype 1 and haplotype 2), both with genome assembly sizes similar to the estimated value from GenomeScope 2.0 (Fig. 1B). Haplotype 1 (mDugDug1.hap1) consists of 198 scaffolds spanning 3.159 Gbp with contig N50 of 57.6 Mbp, scaffold N50 of 140.7 Mbp, longest contig of 162.2 Mbp, and largest scaffold of 267.9 Mbp. Haplotype 2 (mDugDug1.hap2) consists of 167 scaffolds, spanning 3.155 Gbp with contig N50 of 57.9 Mbp, scaffold N50 of 138.0 Mbp, largest contig 209.4 Mbp, and largest scaffold of 230.2 Mbp. Detailed assembly statistics are reported in Table 2, and graphical representation for haplotype 1 in Fig. 1D (Supplementary Fig. 1B for haplotype 2). Haplotype 1 has a BUSCO completeness score of 97.9% using the Vertebrata gene set, a per-base quality (QV) of 70.5, a k-mer completeness of 97.9, and a frameshift indel QV of 41.52; while haplotype 2 has a BUSCO completeness score of 97.8% using the same gene set, a per-base quality (QV) of 70.3, a k-mer completeness of 97.9, and a frameshift indel QV of 42.16.

During manual curation of haplotype 1, we broke 6 joins made by YaHS, closed a total of 23 gaps, and removed one mitochondrial haplotig identified as contamination. The Omni-C contact maps show that both assemblies are highly contiguous; with 24 chromosome-level scaffolds, 23 autosomes, and an X chromosome (Fig. 1C and Supplementary Fig. 1A). We have deposited both assemblies on NCBI (see Table 2 and Data Availability for details).

Final mitochondrial genome size assembled with MitoHiFi was 16,858 bp. The base composition of the final mitochondria assembly is *A* = 30.29%, *C* = 28.60%, *G* = 14.73%, *T* = 26.37%, and consists of 22 unique transfer RNAs and 13 protein-coding genes (Supplementary Fig. 2).

**Fig. 2.**
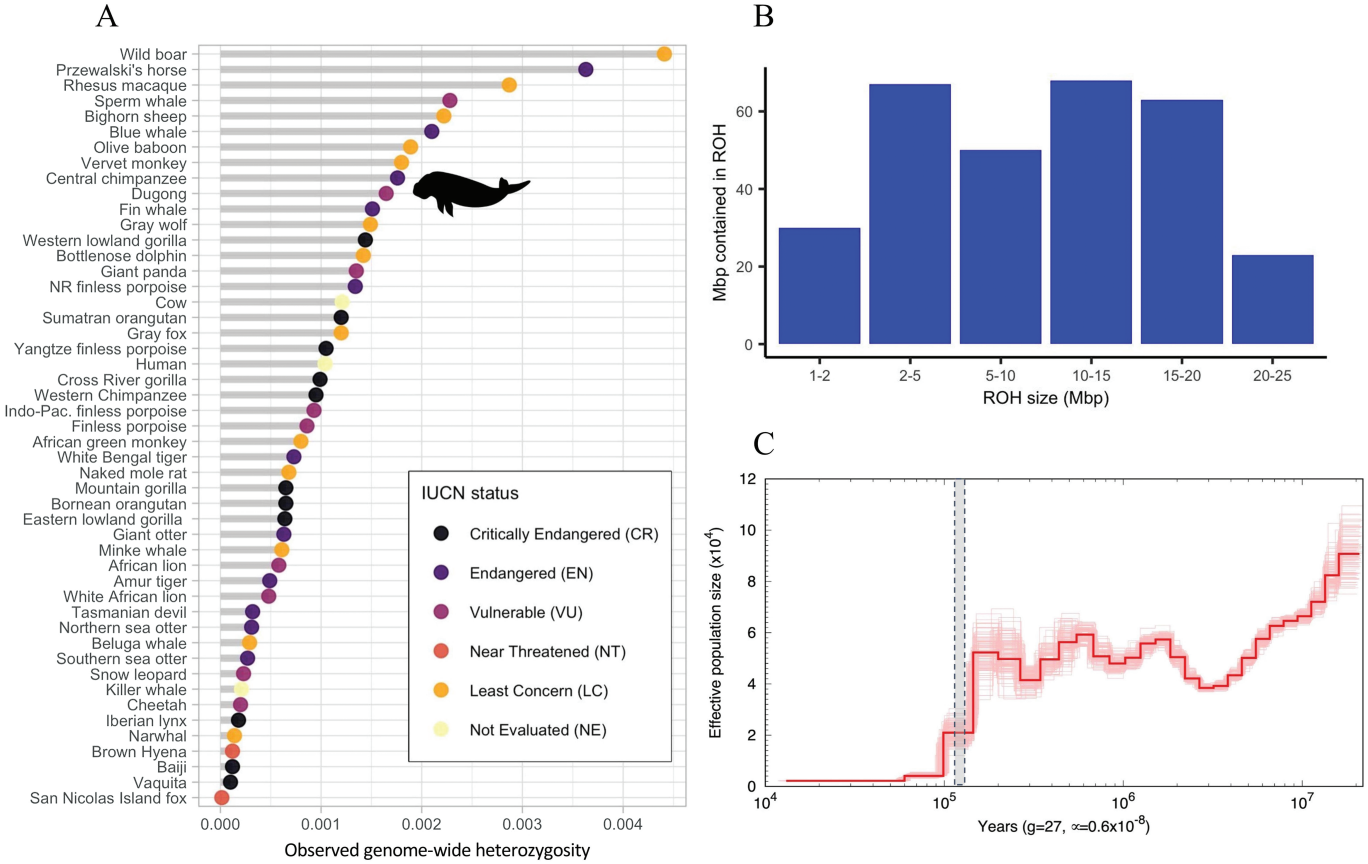
Diversity and demographic history of the dugong. A) Comparison of genome-wide heterozygosity in dugongs and other mammals drawn from the literature, based on Robinson et al. (2016). Dots are colored by the endangered status according to the International Union for Conservation of Nature (IUCN) Red List for Threatened Species. B) Count of runs of homozygosity (ROH) ≥1 Mbp across the dugong autosomal chromosomes of this study, binned by size. C) Effective population size over time, inferred with PSMC and scaled to the dugong generation time and mutation rate. Lighter lines represent bootstrap replicates. Vertical dashed bar represents the last interglacial period from approximately 130 to 115 ka.

We estimated average genome-wide heterozygosity to be 0.165% (0.129% to 0.211%), relatively high for a species of conservation concern (Fig. 2A). Approximately 11% of the genome is in ROH, however, the majority of these are relatively small (<20 Mbp), indicating that most inbreeding did not occur recently (Fig. 2B).

PSMC estimates of effective population size over time indicate that dugong abundance was high (~600,000 individuals) prior to the last interglacial period ~100 ka (1,000 yr ago) but underwent several fluctuations before declining steeply ~100 ka (Fig. 2C).

## Discussion

We present a draft genome assembly for the culturally important dugong, assembled using long reads, and chromosome-scale sequencing data. Genome assemblies are available on NCBI for two other Sirenians, the Florida subspecies of the West Indian manatee (*Trichechus manatus*) (GenBank Assemblies: GCA_000243295.1 and GCA_030013775.1) and the extinct Steller’s sea cow (GenBank assembly: GCA_013391785.1), as well as two previous de novo assemblies for the dugong (GenBank assemblies: GCA_905400935.1 and GCA_905400935.1). No genomic data has been published for the Amazonian (*Trichechus inunguis*) or West African (*Trichechus senegalensis*) manatee species, both of which are listed as Vulnerable by the IUCN. Our assembly is the most contiguous sirenian genome assembly to date, improving on previous assemblies—all assembled with short-read data—by at least an order of magnitude in contigs and scaffold N50s.

Initial estimates of genome-wide heterozygosity based on our new genome assembly are relatively high for a mammal of conservation concern (Fig. 2A), probably reflecting the previously high abundance of dugongs prior to the last interglacial period (ca. 125,000 yr ago). While ROH indicate past inbreeding, we find no evidence in the genome of ongoing inbreeding among the Moreton Bay population of dugongs where this reference individual was sourced from. Future analyses of individuals from different populations may show whether these patterns of diversity are replicated in smaller and more isolated populations.

Our demographic inference analysis based on PSMC suggests that dugongs in Eastern Australia were variably abundant from around 1 million yr ago (Ma) to 150 ka. This earlier estimate coincides with the mid-Pleistocene transition, during which longer and more intense glacial cycling began. However, more recent fluctuations in dugong abundance do not precisely track the approximately 100 ka glacial cycles that drove changes in global sea level (Yehudai et al. 2021). Dugong abundance declined steeply beginning at ~100 ka, probably due to population fragmentation (Blair et al. 2014) and habitat loss that occurred as sea levels fell after the last interglacial period and the shallow seagrass meadows in which they lived disappeared.

Our draft genome assembly promises to advance understanding of marine mammal evolution and diversification as well as provide crucial insights into dugong conservation and management. Sirenians are the most ancient lineage of marine mammals, having split from their most recent terrestrial ancestor ~63.9 Ma (Yuan et al. 2021). Future comparative genomic studies both within Sirenia and between sirenians and other marine mammal lineages will shed light on the genomic changes that allowed for these lineages to adapt to the marine environment. For example, a more contiguous dugong reference genome will improve reference-guided assembly of the extinct Steller’s sea cow, which was notable for both its large size and its adaptation to a subpolar kelp forest environment, unique among the typically warm water dwelling Sirenia. Future generation of genome data from other dugong populations, many of which are geographically isolated and/or live in quite different environments, will allow evolutionary analyses of adaptations unique to this lineage. The species’ large but discontinuous geographic range raises the possibility that some populations are genetically distinct and locally adapted. By identifying isolated populations and better-defining subpopulation units, future work will allow development of more targeted management strategies that can support the continued persistence of this unique marine mammal in changing global habitats.

## Supplementary Material

esae003_suppl_Supplementary_Figures_S1-S2

## Data Availability

Data generated for this study are available under NCBI BioProject PRJNA970804. Raw PacBio HiFi and Omni-C Illumina sequencing data for NCBI BioSample SAMN33212336 are available at https://genomeark.s3.amazonaws.com/index.html?prefix=species/Dugong_dugon/mDugDug1/ge nomic_data/, pending submission to the NCBI Short Read Archive (SRA). GenBank accessions for both primary and alternate assemblies are GCA_030035585.1 and GCA_030020955.1. The mitochondrial genome is available at https://genomeark.s3.amazonaws.com/index.html?prefix=species/Dugong_dugon/mDugDug1/as sembly_MT_rockefeller/ pending submission to GenBank. Assembly scripts and other data for the analyses presented can be found at the VGP galaxy project: https://galaxyproject.org/projects/vgp/.
