# Supplementary material for: A chromosome-level genome assembly for the dugong (Dugong dugon)
Source: J Hered. 2024 Jan 20;115(2):212–20. doi: 10.1093/jhered/esae003 (PMC10936554; doi:10.1093/jhered/esae003)
Supplement: esae003_suppl_Supplementary_Figures_S1-S2 [file esae003_suppl_supplementary_figures_s1-s2.docx]

Supplementary figures


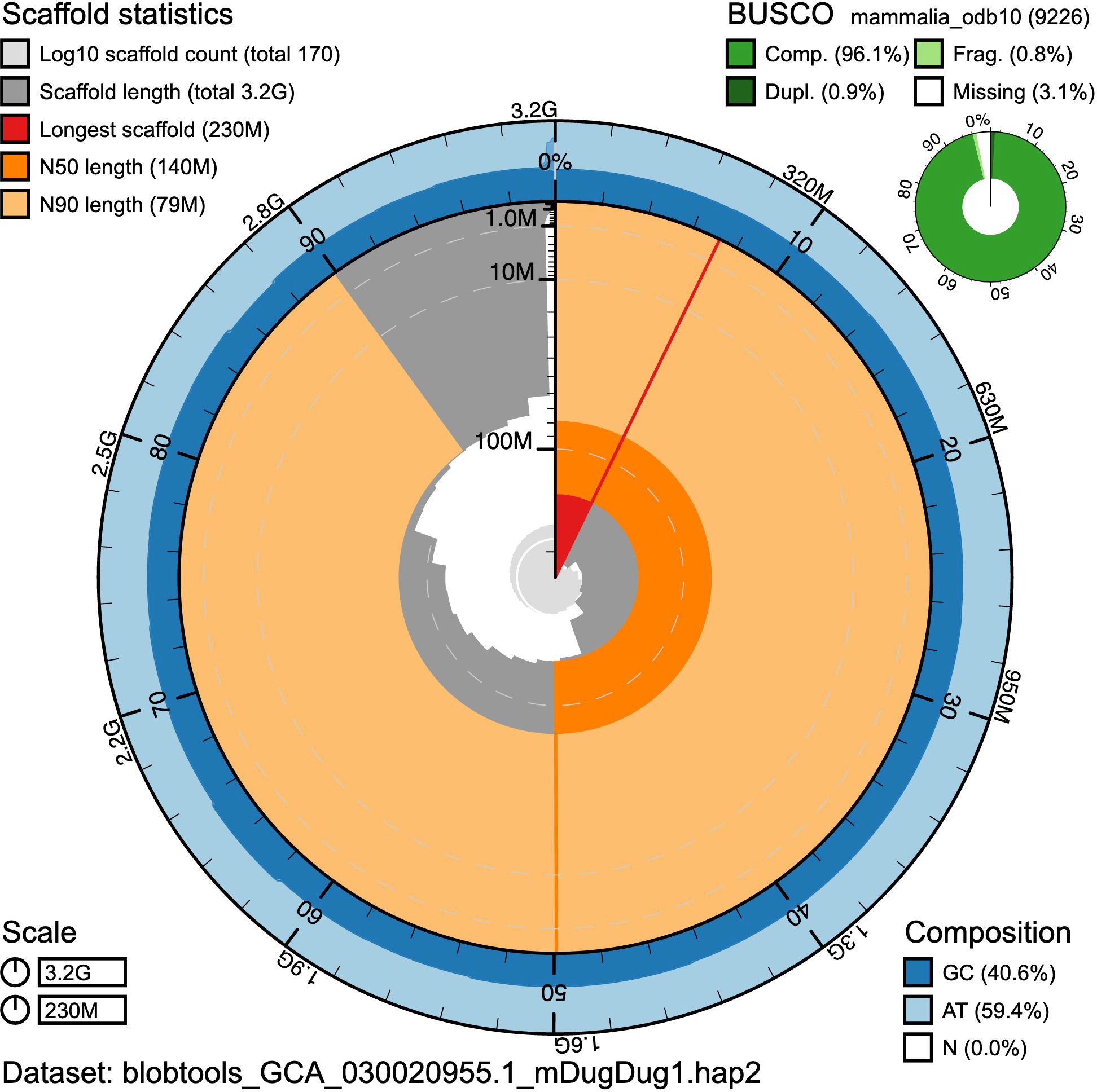
A B

Genomic position


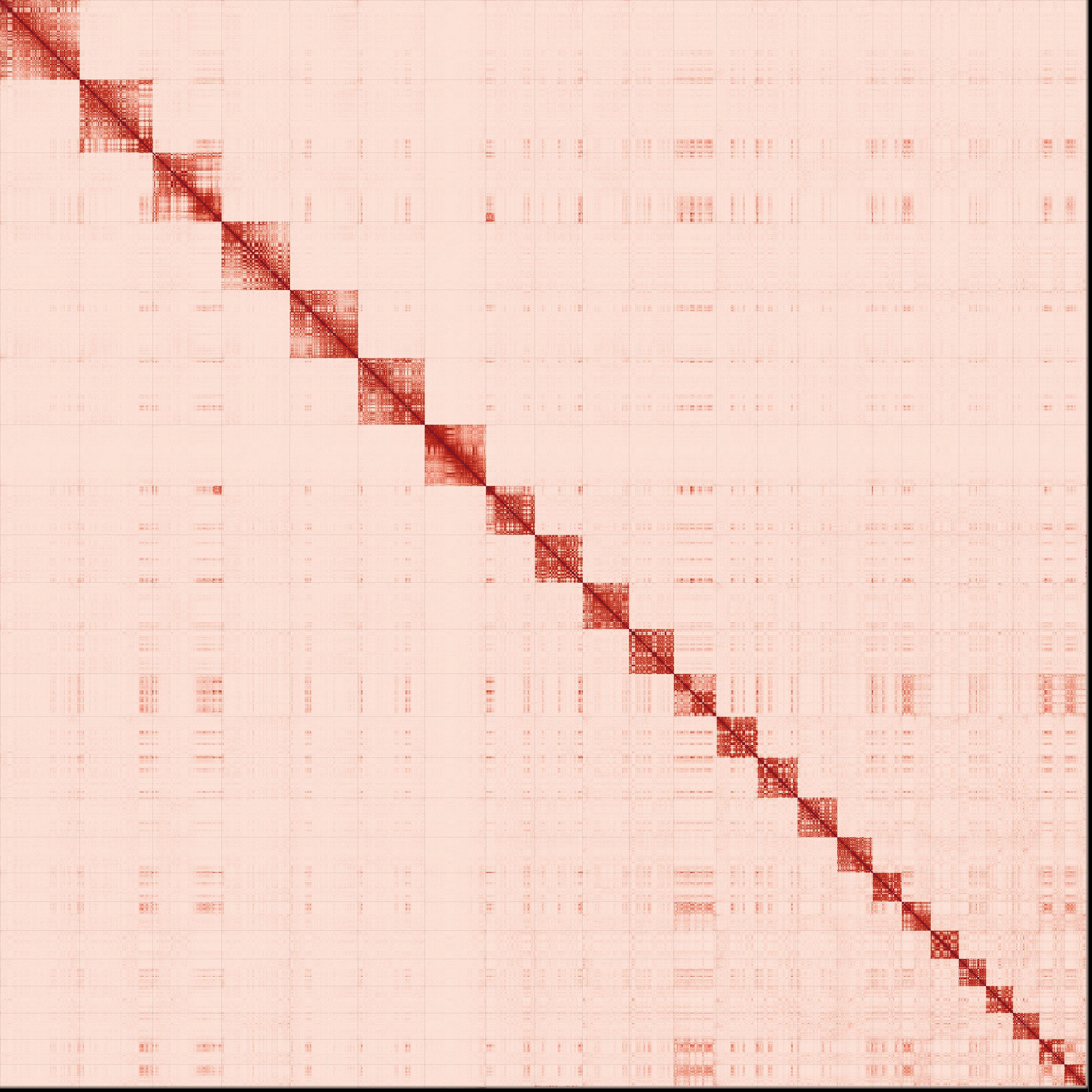


Genomic position

**Supplementary figure 1:** Visual overview of haplotype 2 genome assembly metrics. (A) Omni-C Contact maps for the haplotype 2 genome assembly generated with PretextSnapshot. (B) BlobToolKit Snail plot showing a graphical representation of the quality metrics presented in Table 2 for the *Dugong dugong* haplotype 2 assembly (mDugdug1.hap2) (full description in Fig. 1).


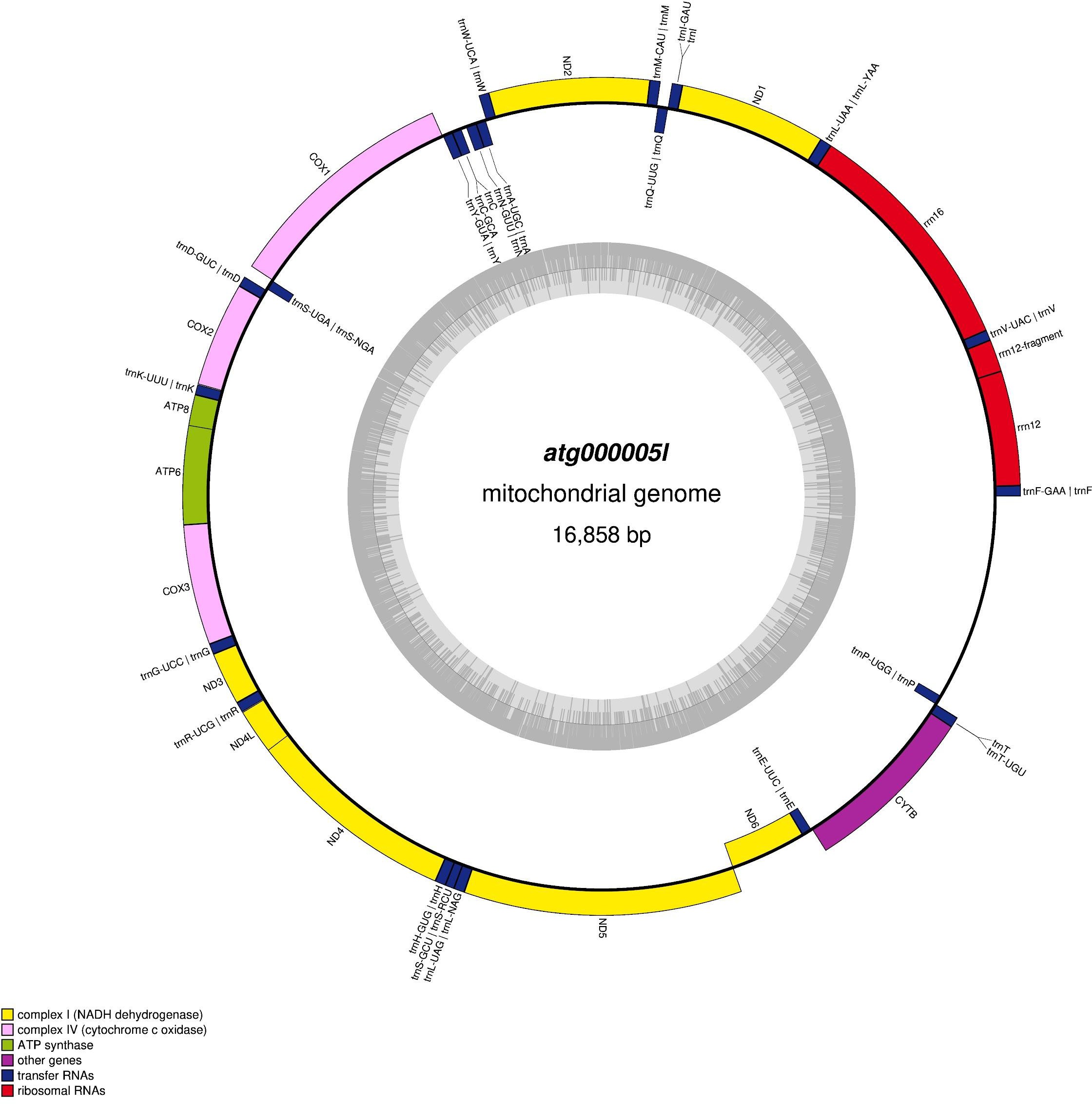


**Supplementary figure 2:** Visual overview of mitochondrial genome assembly annotation. Inner circle represents GC content, with circle marking 50% threshold.
